# The Disruptions of Sphingolipid and Sterol Metabolism in the Short Fiber of *Ligon-Lintless-1* Mutant Revealed Obesity Impeded Cotton Fiber Elongation and Secondary Cell Wall Deposition

**DOI:** 10.3390/ijms26031375

**Published:** 2025-02-06

**Authors:** Huidan Tian, Qiaoling Wang, Xingying Yan, Hongju Zhang, Zheng Chen, Caixia Ma, Qian Meng, Fan Xu, Ming Luo

**Affiliations:** Engineering Research Center of South Upland Agriculture, Ministry of Education, Southwest University, Chongqing 400000, China; thd199834@163.com (H.T.); wql19980513@163.com (Q.W.); yxingying@swu.edu.cn (X.Y.); juju_106@163.com (H.Z.); hero666666c@163.com (Z.C.); mcx2116@email.swu.edu.cn (C.M.); mqhongbin@foxmail.com (Q.M.); xufanfeiren@163.com (F.X.)

**Keywords:** cotton fiber length, lipid, steryl ester, GIPC, lipidomics, oil body

## Abstract

Boosting evidence indicated lipids play important roles in plants. To explore lipid function in cotton fiber development, the lipid composition and content were detected by untargeted and targeted lipidomics. Compared with rapid elongation fibers, the lipid intensity of 16 sub-classes and 56 molecular species decreased, while only 7 sub-classes and 26 molecular species increased in the fibers at the stage of secondary cell wall deposition. Unexpectedly, at the rapid elongation stage, 20 sub-classes and 60 molecular species increased significantly, while only 5 sub-classes and 8 molecular species decreased in the *ligon lintless-1* (*li-1*) mutant compared with its wild-type Texas Maker-1 (TM-1). Particularly, campesteryl, sitosteryl, and total steryl ester increased by 21.8-, 48.7-, and 45.5-fold in the *li-1* fibers, respectively. All the molecular species of sphingosine-1-P, phytoceramide-OHFA, and glucosylceramide increased while all sphingosine, phytosphingosine, and glycosyl inositol phospho ceramides decreased in the *li-1* fibers. Similarly, the different expression genes between the mutant and wild type were enriched in many pathways involved in the lipid metabolism. Furthermore, the number of lipid droplets also increased in the *li-1* leaf and fiber cells when compared with the wild type. These results illuminated that fiber cell elongation being blocked in the *li-1* mutant was not due to a lack of lipids, but rather lipid over-accumulation (obesity), which may result from the disruption of sphingolipid and sterol metabolism. This study provides a new perspective for further studying the regulatory mechanisms of fiber development.

## 1. Introduction

Cotton is an important natural fiber crop in the world. Cotton fiber is the major economic product of cotton and a predominant material in the textile industry. Fiber is a single cell derived from the epidermis of the outer integument of the cotton ovule with extreme elongation and significant thickening on the SCW (secondary cell wall) [1].The growth and development of fiber can be divided into four distinct and overlapping stages: initiation, elongation (primary wall formation), SCW deposition, and maturation. On the day of anthesis, a large number of globular protrusions were observed on the ovule surface, followed by polar elongation. Around 10 DPA (days post-anthesis), the fiber elongation rate reaches the peak, which is the rapid elongation stage of fibers [1,2,3]. Fiber elongation lasts until 16–20 DPA. After about 15 DPA, fiber elongation gradually stops followed by the synthesis of the secondary cell wall, a stage which is also called the transition period from primary wall synthesis to secondary wall synthesis or the initiation of secondary wall synthesis [3]. After 20 DPA, the elongation of the fiber cells stopped completely, and the cells entered into the stage of SCW deposition [3]. The elongation and SCW synthesis stages are two important stages of fiber development, which determine fiber quality (length, strength, and fineness). Since the fiber is a single cell with an extremely elongated (final length up to 30–40 mm) and strikingly thickened secondary wall (cellulose content >90%), and the two actions last for a long period, it is regarded as an ideal material for studying cell elongation, cellulose synthesis, and SCW deposition [2,3,4,5]. However, the regulatory mechanism of cotton fiber cell elongation and SCW formation still needs further exploration.

Cotton fiber mutants are a powerful resource to study the regulatory mechanism of fiber cell development owing to the morphological and biochemical variances in their fiber cells. *Ligon lintless* (*li-1*) is a simply inherited, monogenic dominant mutant characterized by very short and thick fibers (about 6 mm in length) [6,7]. This presents an excellent model system for studying the underlying molecular and cellular processes involved in cotton fiber elongation [8,9,10,11,12]. The fiber initiation and early elongation of *li-1* was similar to that of its wild-type TM-1 (Texas marker-1). At 7 DPA, fiber elongation was inhibited and stopped completely at 14 DPA. During 7–14 DPA, the fiber elongation rate of the *li-1* mutant was much lower than that of the wild type in the same period [12,13,14].

Lipids are essential components of all plant cells, not only as the main component of cell membranes, but also as an important energy source and quality indicator [15,16]. They are involved in the regulation of various life processes, such as transport, signaling, energy conversion, cell development and differentiation, and apoptosis [17]. In plants, fatty acid signaling plays a crucial role in defense and development. These studies are of great significance to basic biology and agriculture. In both rapid elongation and SCW deposition, lipid synthesis is required for cotton fiber cell development [18]. Previous studies reported that the transcript of genes involved in lipid synthesis such as fatty acid desaturase, acyl carrier protein, glycerol-3-phosphate acyltransferase, acyltransferase, lipid transfer proteins, and elongase are significantly enriched in fibers [18,19,20,21,22,23,24,25]. Furthermore, a few studies have been conducted by metabolomics. Glycerides were detected in fibers and showed that polar lipid phosphatidylglycerol including PC (phosphatidylserine), PE (phosphatidylcholine), PI (phosphatidylinositol), PA (phosphatidic), monogalactosyldiacylglycerol (MGDG), and digalactosyldiacylglycerol (DGDG) had the highest content in elongation fibers (7–10 DPA) [26]. Six glycerophospholipids (GPLs) were detected in the wild-type fast elongation fibers and ovules, as well as the *lintless*–*fuzzless* mutant ovules by targeted lipidomics. Phosphatidylinositol (PI) (containing the linolenic acid group) was significantly accumulated in the elongating fibers, indicating that PI plays a role in the elongation of fibers [18]. The content of saturated VLCFA (very-long-chain fatty acids) in elongating fibers is significantly higher than that in ovules and *lintless*–*fuzzless* mutant ovules. The exogenous application of ACE, a fatty acid synthesis inhibitor, inhibited fiber elongation, while VLCFA promoted fiber elongation by induced ethylene synthesis [23]. By analyzing the differences in metabonomics between the elongation stage and the secondary wall synthesis stage of fibers, the result showed that the lipid metabolism was active in the fiber elongation stage [22]. These studies indicated that lipid metabolism plays important roles in the elongation and secondary wall synthesis of fibers.

Sphingolipids are complex lipids that consist of three main components, long-chain fatty acids (LCFAs) or the very-long-chain fatty acids (VLCFAs), the long-chain base (LCB) of sphingosine, and the polar head group [27,28,29]. Recently, a few documents revealed sphingolipid was essential for fiber growth and development. The exogenous application of FB1 (Fumonisin B1), a sphingolipid synthesis inhibitor, strongly inhibited fiber elongation and altered the activity of lipid raft activity in fiber cells [30,31,32]. One kind of phytoceramide molecules containing hydroxylated and saturated VLCFA is important for fiber cell elongation [33]. The contents of all GluCer (glycosylceramides) and GIPC (glycosyl inositol phospho ceramides) molecular species were decreased in 0-DPA ovules of *Xuzhou142 lintless*–*fuzzless* mutants and *Xinxiangxiaoji lintless*–*fuzzless* mutants when compared with the wild-type Xuzhou142 [34]. Overexpressing *GhCS1*, a ceramide synthase gene, inhibited fiber cell initiation and elongation [35]. Since VLCFA is a composition of sphingolipid molecules, downregulating *GhKCRL1*, a gene involved in the VLCFA biosynthesis pathway blocked sphingolipid synthesis and suppressed fiber cell elongation [36]. Regulating GhLCBK1, a sphingosine kinase in cotton, could regulate fiber elongation and SCW deposition through affecting sphingosine-1-phophate and auxin synthesis [37]. These reports indicated that sphingolipids play important roles in fiber cell development. However, the sphingolipid profile in the short fibers of the *li-1* mutant is unknown.

High-throughput lipid mass spectrometry is used to track metabolic changes and rapidly analyze the changes in individual lipid molecules in the wild type and mutant as well as various developmental stages. The untargeted lipidomics model can realize the systematic analysis of various types of lipids in the sample without bias. In order to clarify the lipid differences between the rapid elongation stage (10 DPA) and secondary cell wall synthesis stage (20 DPA) of fibers, as well as the lipid differences between the short fibers of the *li-1* mutant and its wild-type normal fibers at the rapid elongation stage (10 DPA), and to reveal the function of lipids in fiber development, firstly, we analyzed the lipid changes in the 10-DPA and 20-DPA fiber cells of wild-type (TM-1), as well as the lipid differences in 10-DPA fibers between the TM-1 and *li-1* mutant, through untargeted lipidomics assay. On this basis, further analysis was conducted on the differences in sphingolipids and sterols in 10-DPA fibers between the TM-1 and *li-1* mutant through targeted lipidomics assay. The results indicated that the disruptions of sphingolipid and sterol metabolism may be an important cause for the hindered elongation of *li-1* short fibers. This study provides a new clue for further analyzing the regulatory mechanisms of fiber growth and development.

## 2. Results

### 2.1. Untargeted Lipidomics Analysis in Fiber Cells

**Figure 1:**
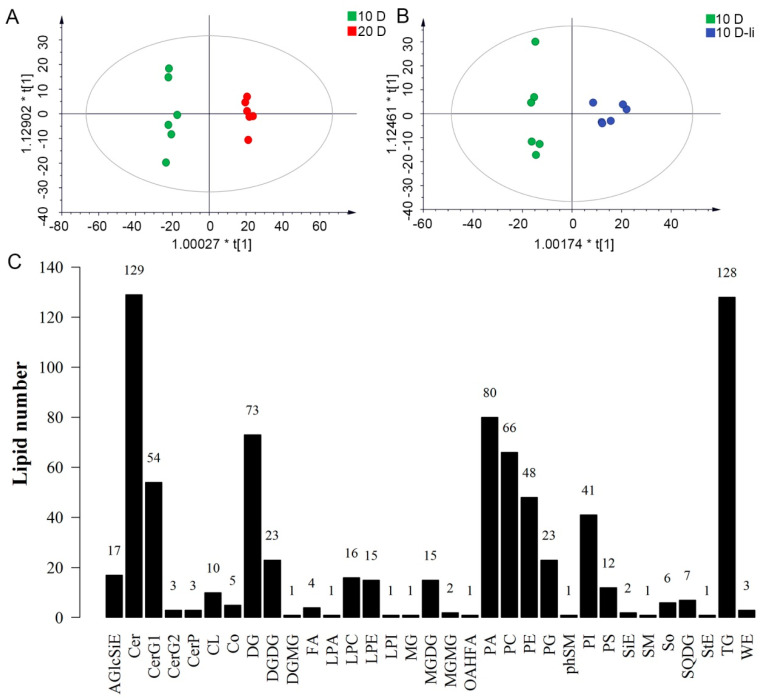
OPLS-DA score plot and the number of lipid species in each detected lipid class. (**A**): The OPLS-DA score plot between the 10-DPA fiber group and 20-DPA fiber group, * represents the multiplication sign. (**B**): The OPLS-DA score plot between the 10-DPA fiber group of wild type (TM-1) and the 10-DPA fiber group of li-1 mutant. (**C**): Thirty-three lipid classes detected in three samples and the number of lipid molecule species in each detected lipid class. The number on each column represents the number of molecular species in each lipid class. 10 D: 10-DPA fibers of TM-1 (wild type); 20 D: 20-DPA fibers of TM-1 (wild type); 10 D-li: 10-DPA fibers of *li-1* mutant. AGlcSiE, AcylGlcSitosterol ester; Cer, ceramides; CerG1, glucocerebroside; CerP, ceramides phosphate; CL, cardiolipin; Co, coenzyme; DG, diglyceride; DGMG, Digalactosylmonoacylglycerol; DGDG, digalactosyldiacylglycerol; FA, fatty acid; LPA, lysophosphatidic acid; LPC, lysophosphatidylcholine; LPE, lysophosphatidylethanolamine; LPI, lysophosphatidylinositol; MG, monoglyceride; MGDG, monogalactosyldiacylglycerol; MGMG, Monogalactosylmonoacylglycerol; OAHFA, O-Acetylated Hydroxy Fatty Acid. PA, phosphatidic acid; PC, phosphatidylserine; PE, phosphatidylcholine; PG, phosphatidylglycerol; phSM, phytosphingosine; PI, phosphatidylinositol; PS, phosphatidylserine; SiE, sitosterol ester; SM, sphingomyelin; So, sphingosine; MG, monoglyceride; SQDG, Sulfoquinovosyldiacylglycerol; StE, stigmasterol ester; TG, triglyceride; WE, wax esters.

To identify the lipid differences in cotton fiber cells at the rapid elongation stage and secondary wall synthesis stage, as well as the lipid differences in fibers of the *li-1* mutant, the OPLS-DA (Orthogonal Partial Least Squares Discriminant Analysis) model was used. Both R2Y and Q2 are the evaluation parameters of the model. As shown in the score plot (Figure 1A,B), there were six scores in each group and two groups were clearly separated. In the model, R^2^Y = 0.994 and Q^2^ = 0.980 in the group of the 10-DPA fibers and 20-DPA fibers of the wild type, and R^2^Y = 0.949 and Q^2^ = 0.870 in the group of the 10-DPA fibers of the *li-1* mutant and 10-DPA fibers of the wild type, indicating that the quality of the OPLS-DA model was excellent to screen the key lipids between two samples.

**Figure 2:**
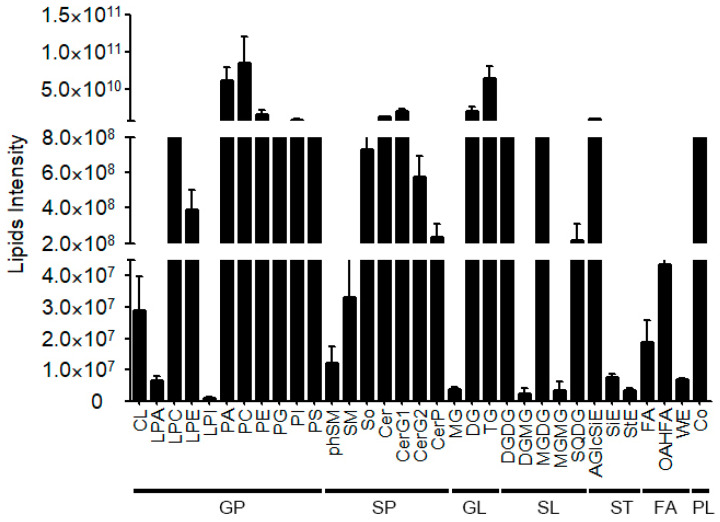
The lipid intensity of various lipid classes. The intensity of 7 classes of lipids including GP, SP, GL, SL, ST, FA, and PL and 33 kinds of lipid sub-classes in the fiber cell. GP, glycerophospholipid; SP, sphingolipid; GL, glycerolipid; ST, sterol lipids; PL, prenol lipid; FA, fatty acid; SL, saccharolipid; AGlcSiE, AcylGlcSitosterol ester; Cer, ceramides; CerG1, glucocerebroside; CerP, ceramides phosphate; DG, diglyceride; CL, cardiolipin; DGMG, Digalactosylmonoacylglycerol; DGDG, digalactosyldiacylglycerol; LPA, lysophosphatidic acid; LPC, lysophosphatidylcholine; LPE, lysophosphatidylethanolamine; LPI, lysophosphatidylinositol; MG, monoglyceride; MGDG, monogalactosyldiacylglycerol; MGMG, Monogalactosylmonoacylglycerol; OAHFA, O-Acetylated Hydroxy Fatty Acid; PA, phosphatidic acid; PC, phosphatidylserine; PE, phosphatidylcholine; PG, phosphatidylglycerol; phSM, phytosphingosine; PI, phosphatidylinositol; PS, phosphatidylserine; SiE, sitosterol ester; SM, sphingomyelin; So, sphingosine; SQDG, Sulfoquinovosyldiacylglycerol; StE, stigmasterol ester; TG, triglyceride; WE, wax esters; Co, coenzyme.

In total, 7 lipid classes (glycospholipids, sphingolipids, glycolipids, sterol esters, prenol lipids, fatty acyls, and saccharolipids) were identified in fiber cells including 33 lipid sub-classes and 793 lipid molecular species (Figure 1C). Sphingolipids comprised seven lipid sub-classes and 197 lipid molecule species (129 ceramides, 3 phosphoceramides, 6 sphingosine, 1 phytosphingosine, 1 sphingomyelin, 54 glucosylceramides, and 3 disaccharide ceramides). Glycerophospholipids included 11 lipid sub-classes and 313 lipid molecule species; the number of molecular species in each sub-class was 10 CL, 1 LPA, 16 LPC, 15 LPE, 1 LPI, 80 PA, 66 PC, 48 PE, 23 PG, 41 PI, and 12 PS. Glycerolipids had three lipid sub-classes: MG, DG, and TG; the number of molecular species was 1, 73, and 128, respectively. Three lipid sub-classes of fatty acyls were detected such as FA, OAHFA, and WE; the number of molecular species was 4, 1, and 3, respectively. Five lipid sub-classes of saccharolipids, DGDG, DGMG, MGDG, SQDG, and MGMG, were detected; the number of molecular species was 23, 1, 15, 7, and 2, respectively. Sterol esters included three lipid sub-classes: AGlcSiE, SiE, and StE; the number of molecular species was 17, 2, and 1, respectively. One lipid class Co (comprised 5 lipid molecular species) was detected in fiber cells (Figure 1C). In the 10-DPA fiber cells, the lipid sub-classes PA, PC, PE, PI, Cer, CerG1, DG, TG, and AGlcSiE possessed a higher lipid intensity while the lipid sub-classes LPI, Mg, DGMG, MGMG, and StE had a lower lipid intensity (Figure 2 and Data S1). The results indicated that glycerophspholipid, glycerolipid, and sphingolipid are predominant lipids and contained more kinds of lipid molecule species in fiber cells.

### 2.2. The Lipid Difference Between the Stage of Rapid Elongation and SCW Deposition of Fiber Cells

**Figure 3:**
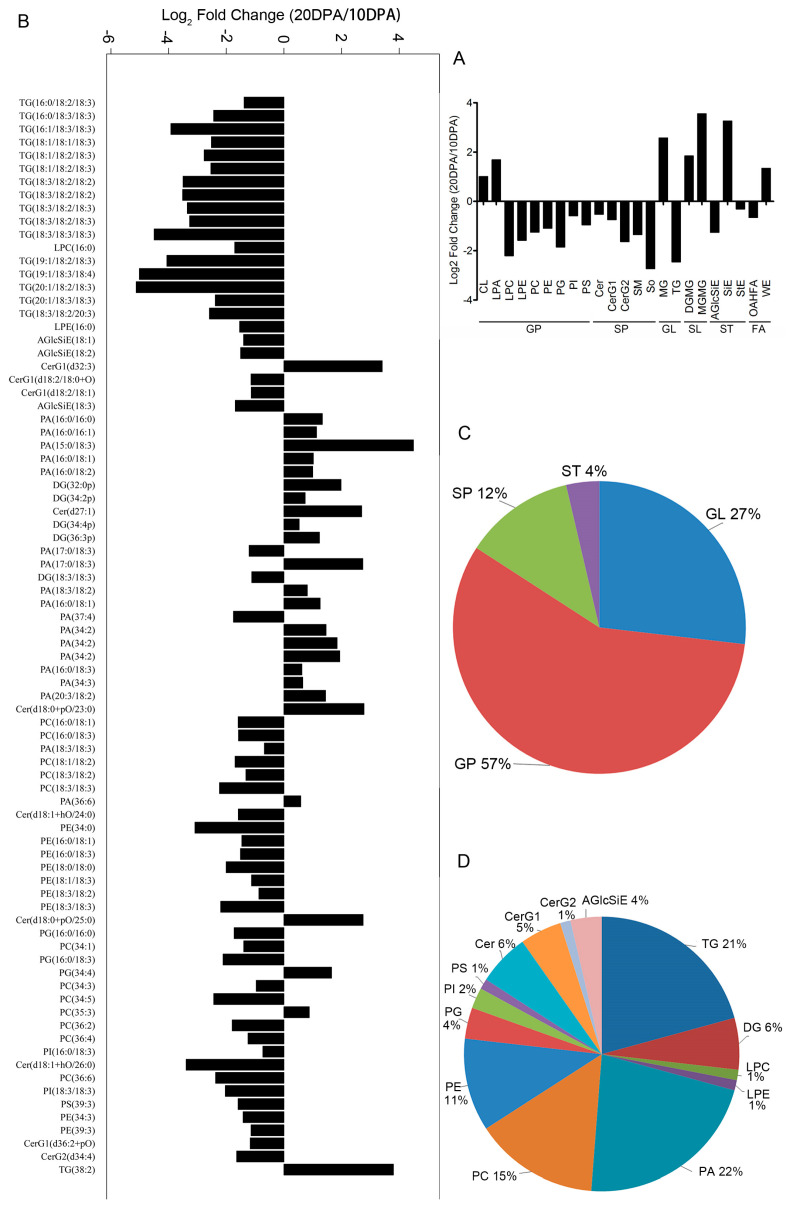
The intensity difference in lipid sub-classes and lipid molecule species between 10-DPA fiber cells and 20-DPA fiber cells. (**A**): The fold changes in various lipid sub-classes between 10-DPA fiber cells and 20-DPA fiber cells; (**B**): the fold changes in various lipid molecule species between 10-DPA fiber cells and 20-DPA fiber cells; (**C**): the proportion of various lipid compounds in the total significantly changed lipids; (**D**): the proportion of various lipid molecule species in the total significantly changed lipids. 10 DPA: 10-DPA fibers of TM-1 (wild type); 20 DPA: 20-DPA fibers of TM-1 (wild type).

The rapid elongation period of fiber cells is around 10 DPA. After 15 DPA, the SCW of fiber begins to synthesize and a large amount of cellulose is deposited, while cell elongation stops gradually. Around 20 DPA, fiber cells ceased elongation completely. In order to reveal the lipid difference in various developmental stages of fiber, we analyzed the lipid difference between the 10-DPA fibers and 20-DPA fibers from TM-1. The results are shown in (Figure 3A). Compared with the 10-DPA fibers, 13 lipid sub-classes increased and 20 lipid sub-classes decreased in the 20-DPA fibers. By statistical analysis, 23 lipid classes changed significantly (*p* < 0.05). Among them, seven lipid sub-classes increased such as MGMG, SiE, MG, and so on. Meanwhile, 16 lipid sub-classes decreased and the top 5 lipid sub-classes with the most decrease were So, TG, LPC, PG, and CerG2. The results indicated that the lipid intensity of various lipid classes significantly changed between the rapid elongation and SCW synthesis stages, and the lipids were majorly enriched in the rapid elongation fiber cells.

The detailed lipid profile was further analyzed (Data S2). Compared with the 10-DPA fibers, 82 lipid molecule species significantly changed in the 20-DPA fibers (VIP > 1 and *p* < 0.05). Among them, the lipid intensity of 26 lipid molecule species increased and 56 lipid molecule species decreased (Figure 3B and Figure S1). The top five lipid molecule species with the largest increase in lipid intensity were PA (15:0/18:3), TG (38:2), CerG1 (d32:3), CER (d18:0/23:0), and CER (d18:0/25:0), and their increase folds were 22.54, 13.92, 10.63, 6.87, and 6.72, respectively. The top five lipid molecule species with the largest decrease folds were TG (20:1/18:2/18:3), TG (19:1/18:3/18:4), TG (18:3/18:3), TG (19:1/18:2/18:3), and TG (16:1/18:3/18:3), which decreased by 34.48-, 32.26-, 22.73-, 16.67-, and 15.15-fold, respectively (Figure 3B). The lipid molecule species with a significant change in lipid intensity mainly belonged to glycerophspholipid (57%), glycerolipid (27%), and sphingolipid (12%), and were mainly involved in the lipid sub-classes of PA (22%), TG (21%), PC (15%), and PE (11%) (Figure 3C,D). The results indicated that most lipid molecule species of GP, GL, and SP were strikingly enriched in the rapid elongation stage, and a few lipid molecule species were enriched at the stage of SCW deposition, which might play some roles in SCW formation.

### 2.3. The Lipid Change in Fiber Cells of li-1 Mutant

**Figure 4:**
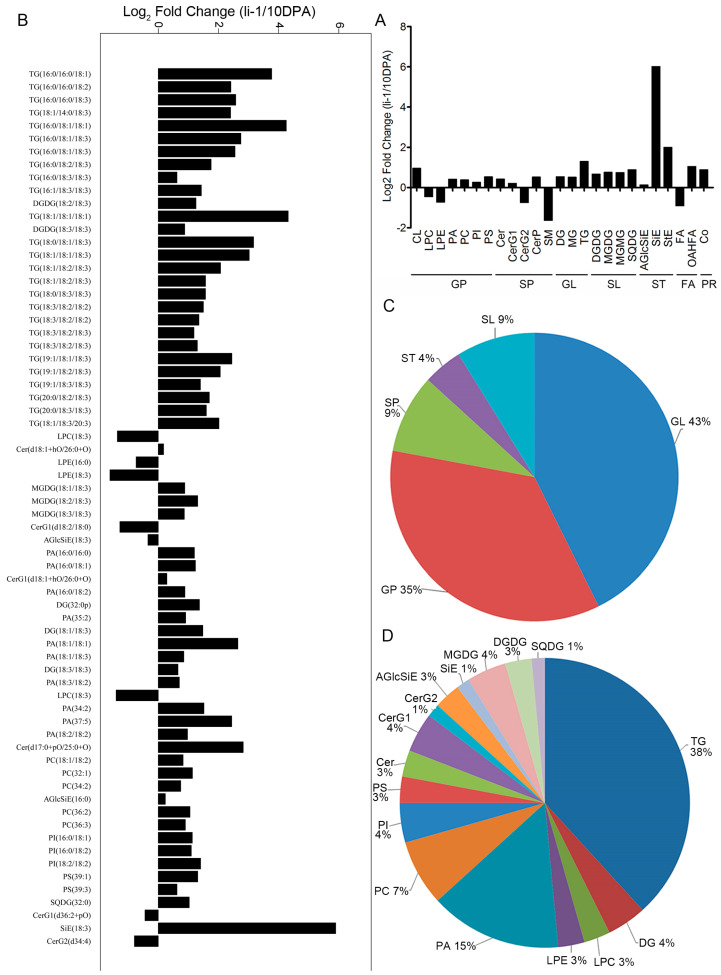
The intensity difference in lipid sub-classes and lipid molecule species between 10-DPA fiber cells of TM-1 (wild type) and 10-DPA fiber cells of *li-1* mutant. (**A**): The fold changes in various lipid sub-classes between 10-DPA fiber cells of TM-1 and 10-DPA fiber cells of *li-1* mutant; (**B**): the fold changes in various lipid molecule species between 10-DPA fiber cells of TM-1 and 10-DPA fiber cells of li-1 mutant; (**C**): the proportion of various lipid compounds in the total significantly changed lipids; (**D**): the proportion of various lipid molecule species in the total significantly changed lipids. 10 DPA: 10-DPA fibers of TM-1 (wild type); 10 *li-1*: 10-DPA fibers of *li-1* mutant.

The *li-1* mutant is a super-short fiber mutant with less than 6 mm length fibers, which is an ideal model for studying fiber development [13]. To explore the role of lipids in cotton fiber elongation, we identified the lipid changes between the *li-1* mutant and its wild type (TM-1) in the 10-DPA fibers. As shown in (Figure 4A), compared with the 10-DPA fibers of TM-1, 23 lipid sub-classes increased and 10 lipid sub-classes decreased in the *li-1* mutant, and 25 of 33 lipid sub-classes changed significantly (*p* < 0.05). Among them, 20 lipid sub-classes increased and the top 5 lipid sub-classes (fold change) with the largest increase were SiE (64.37), StE (3.99), TG (2.46), OAHFA (2.06), and CL (1.94), while only 5 lipid sub-classes decreased, and the top 5 with the largest decrease were SM (3.13), FA (1.85), CerG2 (1.67), LPE (1.64), and LPC (1.37). The results indicated that the lipid intensity of most lipid classes increased significantly in the 10-DPA fiber cells of the li-1 mutant compared with the 10-DPA fiber cells of the wild type.

Compared with the 10-DPA fibers of TM-1, the lipid intensity of 68 lipid molecule species changed significantly in the 10-DPA fibers of the mutant (VIP > 1 and *p* < 0.05). Among them, 60 lipid molecule species increased and 8 decreased (Figure 4B and Figure S2). The top five lipid molecule species with the largest increase were SiE (18:3), TG (18:1/18:1/18:1), TG (16:0/18:1/18:1), TG (16:0/16:0/18:1), and TG (18:0/18:1/18:3), and their increase folds were 59.56, 19.94, 19.10, 13.64, and 8.99, respectively. The top five lipid molecule species with the largest decrease in folds were PLE (18:3), PLC (16:0), PLC (18:3), CerG1 (d18:2/18:0), and CerG2 (d34:4), and their decreased folds were 3.04-, 2.64-, 2.57-, 2.42-, and 1.73-fold, respectively (Figure 4B). The lipid molecule species with a significant change in lipid intensity mainly belonged to glycerolipid (43%) and glycerophspholipid (35%), and mainly involved the lipid sub-classes of TG (38%) and PA (15%) (Figure 4C,D). The results indicated that most lipid molecule species of TG and GP were strikingly enriched in the 10-DPA fibers of the li-1 mutant. Interestingly, the lipid molecule species of SiE (18:3) increased by 59.56-fold, indicating it may be a key factor contributing to short fibers.

### 2.4. The Changes in Sphingolipids in li-1 Mutant Fiber Cells

**Figure 5:**
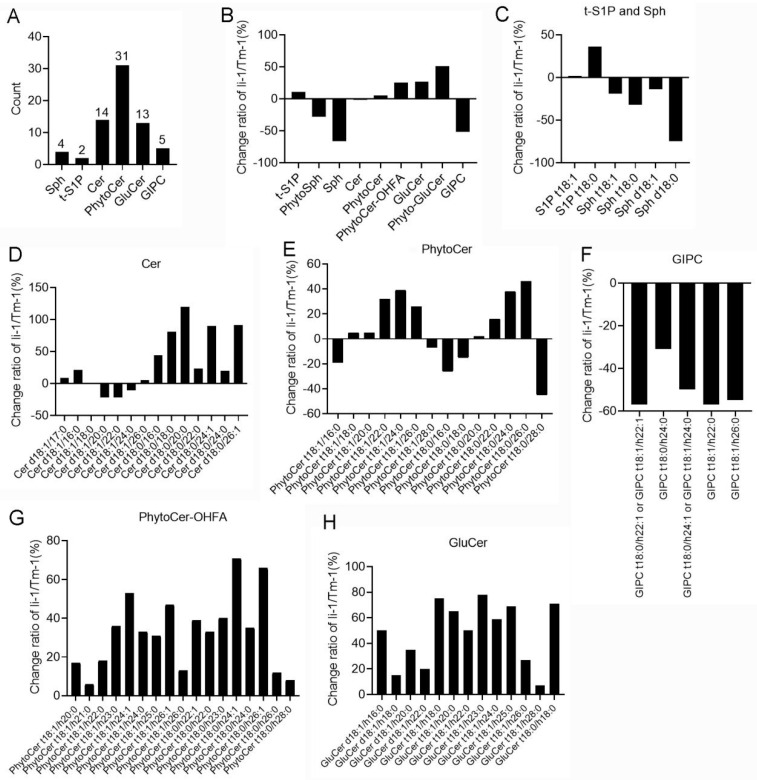
Sphingolipid classes in cotton fiber cells and their alteration in *li-1* mutant fiber cells compared with its wild-type TM-1. The change ratio represents the percentage of increase and decrease in sphingolipid classes and molecular species in *li-1* fiber cells compared to wild-type fiber cells. (**A**): The number of classes and molecular species of sphingolipids detected in fiber cells of *li-1* and TM-1; (**B**): the change percentage of sphingolipid content in *li-1* fiber cells; (**C**): the change percentage of molecular species of t-S1P and Sph; (**D**): the change percentage of molecular species of Cer; (**E**): the change percentage of molecular species of Phyto-Cer; (**F**): the change percentage of molecular species of GIPC; (**G**): the change percentage of molecular species of PhytoCer-OHFA; (**H**): the change percentage of molecular species of GluCer. Cer, ceramides; PhytoCer, phytoceramides; PhytoCer-OHFA, phytoceramides with hydroxylated fatty acyls; S1P, sphingosine-1-phosphate; t-S1P, phytosphingosine-1-phosphate; Sph, sphingosines; PhytoSph, phytosphingosines; GluCer, glucosylceramides; Phyto-GluCer, phyto-glucosylceramides; GIPC, glycosyl inositol phospho ceramides.

The results of the untargeted lipidomics showed significant changes in sphingolipids in the mutant fiber cells compared with the wild-type fibers. To further illuminate the detailed changes in sphingolipid composition and content, we detected the sphingolipid profile of the 10-DPA fiber cells from the *li-1* mutant and TM-1 by means of UHPLC–MS/MS. The results showed that six sub-classes of sphingolipids and 69 molecular species of sphingolipids were detected (Figure 5A), including sphingosines (Sph), sphingosine-1-phosphate (S1P), ceramides (Cer), phytoceramides (PhytoCer), glucosylceramides (GluCer), and glycosyl inositol phospho ceramides (GIPC); the number of molecular species was 4, 2, 14, 31, 13, and 5 for each class, respectively. Compared with the wild type, the content of the Sph (PhytoSph and Sph) and GIPC classes decreased. Meanwhile, PhytoCer-OHFA, GluCer, and Phyto-GluCer increased (Figure 5B). Further analysis revealed that the content of all molecular species of sphingosine and GIPC decreased (Figure 5C,F and Figure S4). On the contrary, the content of all molecular species of GluCer (Phyto-GluCer and GluCer) and PhytoCer OHFA increased (Figure 5G,H and Figure S4). These results indicated that the sphingolipid profile was significantly altered in the *li-1* fibers. It is suggested that GIPC synthesis was impaired, which resulted in all GIPCs decreasing and all GluCer and Cers increasing.

### 2.5. The Changes in Phytosterols in li-1 Mutant Fiber Cells

**Figure 6:**
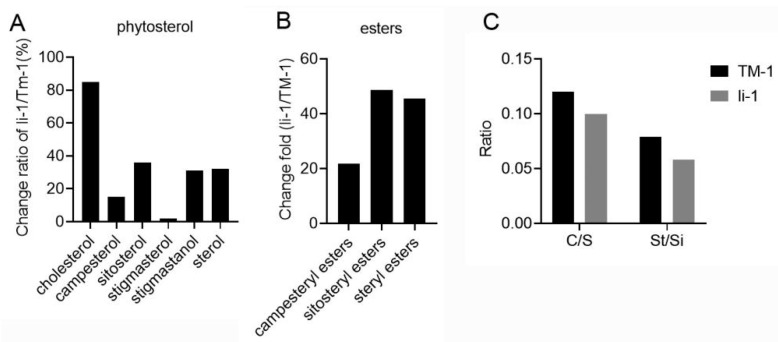
The content changes in sterol and steryl ester in *li-1* fiber cells. (**A**) The content changes in total sterol and various sterol classes in *li-1* fiber cells. (**B**) The content changes in total steryl ester and two steryl esters in *li-1* fiber cells. (**C**) The ratio of stigmasterol to sitosterol (St/Si) and campesterol to sitosterol (C/S) in 10-DPA fibers of mutant and wild type.

In the untargeted lipidomics analysis, the content of sitosteryl ester (SiE) was 59.56 times higher in the *li-1* mutant fibers than in the TM-1 fibers, indicating a striking change in steryl esters occurred in the mutant fiber cells. To further detail the changes in phytosterols in the mutant, we detected the phytosterol profile in both the li-1 and TM-1 10-DPA fibers. Five sterols (cholesterol, sitosterol, campesterol, stigmasterol, and stigmastanol) and two steryl esters were detected in the 10-DPA fiber cells (Figure 6A). Compared with the wild type, the total sterol and all sub-classes of sterols were increased in the mutant fibers, among which, the cholesterol, sitosterol, and stigmastanol were increased by 85%, 36%, and 31%, respectively (Figure 6A). Meanwhile, compared to the wild type, the mutant fiber cells showed a striking increase in campesteryl ester, sitosteryl ester, and total steryl ester, with 21.8-, 48.7-, and 45.5-fold increases, respectively (Figure 6B). Given the proportion between sitosterol, campesterol, and stigmasterol is important for sterol function in plant development, we analyzed the ratio of campesterol to sitosterol (C/S) and stigmasterol to sitosterol (St/Si). The result showed that the ratios of C/S and St/Si were declined in the fibers of the li-1 mutant (Figure 6C), which might result from the content of sitosterol increasing greatly in the li-1 fiber cells. These results indicated that the contents of sterol and steryl ester were altered dramatically in the *li-1* mutant fiber cells.

### 2.6. The Expression of Genes Involved in Lipid Metabolism Was Altered in li-1 Fiber Cells

**Figure 7:**
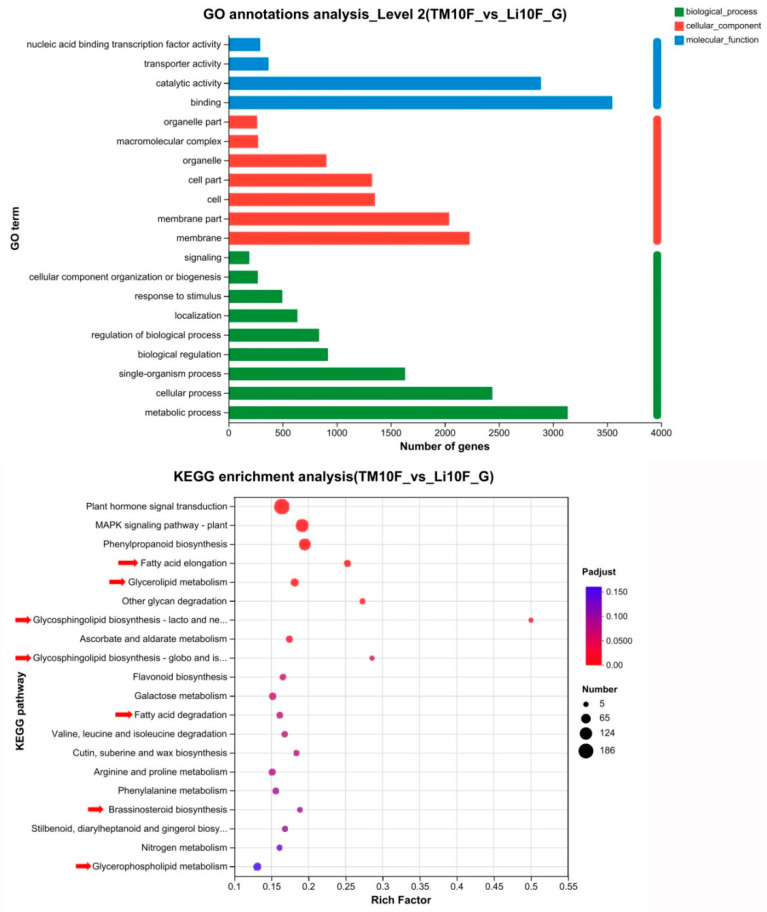
GO annotations and KEGG enrichment analysis for the differentially expressed genes in 10-DPA fiber cells between *li-1* mutant and TM-1 wild type. Red arrows indicated the pathway involved in lipid metabolism.

To clarify the metabolic pathways and key genes that undergo significant changes in the mutant short fiber cells, we analyzed the transcriptome of the 10-DPA fiber cells of the *li-1* mutant and its wild-type TM-1. The results showed significant changes in the expression of 8180 genes in the *li-1* mutant 10-DPA fibers, with 5600 genes upregulated and 3580 genes downregulated (Figure S5). The results of the GO annotations analysis indicated that the differentially expressed genes were enriched in the biological process (catalytic activity and binding), cellular component (membrane part and membrane), and molecular function (cellular process and metabolic process) (Figure 7). The results of the KEGG enrichment analysis revealed that the differentially expressed genes were enriched in lipid metabolism pathways, including fatty acid association and degradation, glycerolipid and glycerophospholipid metabolism, Glycosphingolipid biosynthesis, and Brassinosteroid biosynthesis, 7 out of the top 20 metabolic pathways (Figure 7). These results illuminated that the lipid metabolism was disrupted in the *li*-1 mutant fiber cells compared with the TM-1.

### 2.7. The Expression Levels of Key Genes in Lipid Metabolism Were Elevated in li-1 Fiber Cells

**Figure 8:**
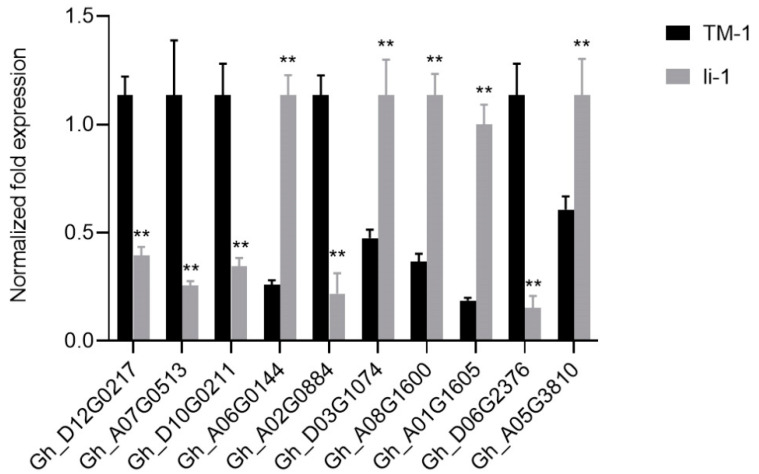
The expression changes in selected genes in *li-1* mutant fiber cells. Gh_D12G0217, LAG1 homologue 2; Gh_A07G0513, LAG1 longevity assurance homolog 3; Gh_D10G0211, Lactosylceramide 4-alpha-galactosyltransferase; Gh_A06G0144, phospholipase A 2A; Gh_A02G0884, GDSL-like Lipase/Acylhydrolase superfamily protein; Gh_D03G1074 and Gh_A05G3810, HXXXD-type acyl-transferase family protein; Gh_A08G1600, phospholipase D beta 1; Gh_A01G1605, alcohol dehydrogenase 1; Gh_D06G2376, 3-ketoacyl-CoA synthase 19. Error bars represent the SD for three independent experiments and asterisks indicate statistically significant differences between *li-1* and TM-1 fiber cells, as determined by Student’s *t*-test (** *p* < 0.01).

To confirm the expression change in genes involved in the lipid metabolism, we detected the expression levels of selected genes in the *li-1* and TM-1 fibers by RT-qPCR. Gh_D12G0217 (LAG1 longevity assurance homolog 2), Gh_A07G0513 (LAG1 longevity assurance homolog 3), and Gh_D10G0211 (Lactosylceramide 4-alpha-galactosyltransferase) were involved in sphingolipid biosynthesis and were downregulated in the *li-1* mutant [35,38]. Gh_A08G1600 and Gh_A06G0144 encoded phospholipase D beta 1 and phospholipase A 2A, respectively. They play a role in phospholipid metabolism and were upregulated in the *li-1* fiber cells. Gh_A02G0884 (GDSL-domain protein) and Gh_D06G2376 (3-ketoacyl-CoA synthase 19) decreased in the *li-1* mutant. Gh_D03G1074 and Gh_A05G3810 encoded two HXXXD-type acyl-transferase family proteins, which were increased in the *li-1* mutant. Gh_A01G1605 (alcohol dehydrogenase 1) might function in fatty acid degradation and was strikingly increased in the *li-1* mutant (Figure 8). The result indicated the expression levels of genes involved in lipid biosynthesis and degradation were changed greatly, suggesting that the lipid metabolism was disrupted in the *li-1* mutant fiber cells.

### 2.8. The Number of Oil Bodies Was Increased in li-1 Leaf and Fiber Cells

**Figure 9:**
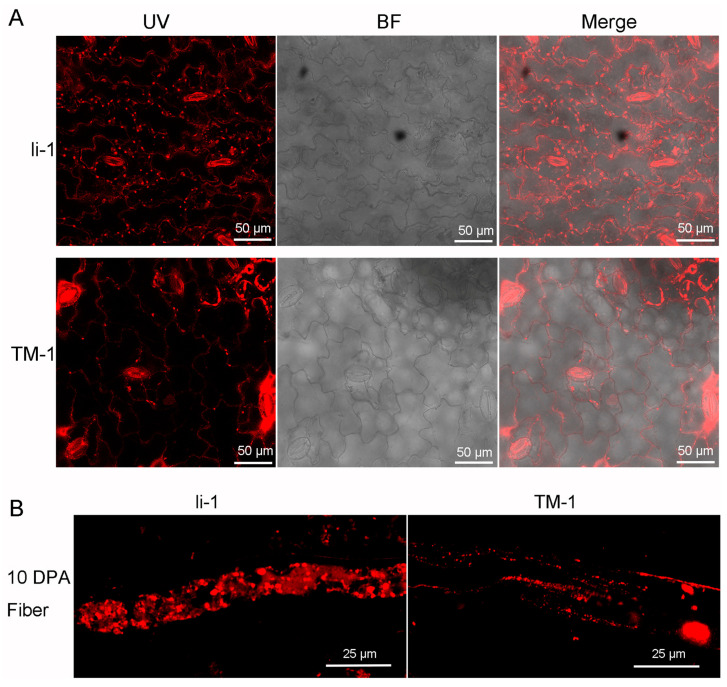
Oil bodies in the leaf and fiber cell of TM-1 and *li-1* mutant. (**A**) Oil bodies in the leaf of TM-1 and *li-1* mutant; (**B**) oil bodies in the fiber cell of TM-1 and *li-1* mutant. *Li-1*, *li-1* mutant; TM-1, wild type; UV, ultraviolet light; BF, bright light.

As mentioned earlier, the content of triglycerides (TG) and steryl esters is far higher in the *li-1* fibers than in the TM-1 fibers. Given that the hydrophobic core of the oil body (lipid droplet) is mainly composed of TG and steryl esters [39,40], we further detected the distribution and quantity of oil bodies in the mutant leaves and fiber cells by Nile red staining. The results showed that only a few small oil bodies were present in the TM-1 leaves and fiber cells while a great number of oil bodies were present in the *li-1* leaves and fiber cells (Figure 9). This result further confirmed that the lipid metabolism was disrupted in the *li-1* mutant.

## 3. Discussion

### 3.1. The Role of Lipids in Fiber Elongation and SCW Deposition

The growth and development of fiber can be divided into four distinct and overlapping stages: initiation, elongation (primary wall formation), SCW deposition, and maturation. Following initiation (0~2 DPA), the fiber cells start to elongate. During the fast elongation period (8~12 DPA), the cell size and membrane area expand rapidly, and various metabolic activities need to be increased accordingly. This process requires a lot of lipids [2,23,30]. At the stage of SCW deposition (15~40 DPA), cell expansion stopped and cellulose synthesis, transportation, and deposition proceeded steadily. The cellulose synthase complex is located in the plasma membrane. The lipid composition and membrane features at this stage are conducive to the synthesis and organization of cellulose [3]. In this study, we analyzed the difference in lipid groups between the fast elongation stage (10 DPA) and the SCW formation stage of fibers (20 DPA). Most of the lipid components (16/23 lipid sub-classes and 56/82 lipid molecular species) were higher in the elongating fibers, and only a few lipid components (7/23 lipid sub-classes and 26/82 lipid molecular species) were higher in the SCW formation stage. Among them, glycerophospholipids (GP), sphingolipids (SP), and glycerolipids (GL) were enriched in the elongated fibers, which was similar to previous studies. It was reported that the content of polar lipids such as PC, PE, PI, PA, and PG was the highest in the elongating fibers (7–10 DPA) [26]. These results suggested that the accumulation of these lipids may be necessary for fiber elongation. The lipids of the 10-DPA fiber and ovule of the wild type, and the 10-DPA ovule of the *fuzzless*–*lintless* mutant were detected by targeted lipidomics. The result showed that phosphatidylinositol (PI) was enriched in fiber cells and PI (34:3) was the highest lipid molecule species [18]. Consistently, in our study, the lipid intensity of PI and molecular species PI (18:3/18:3) and PI (16:0/18:3) molecules was significantly higher in the 10-DPA fiber cells than in the 20-DPA fiber cells of TM-1. The PI intensity of the 10-DPA fibers from the *li-1* mutant was also significantly higher than that of the 10-DPA fibers from the wild-type. However, the increased PI molecules were PI (16:0/18:1), PI (16:0/18:2), and PI (18:2/18:2), which indicated that the PI molecules enriched in the mutant 10-DPA fibers were different from those enriched in the wild-type 10-DPA fibers. Phospholipase D (PLD) can hydrolyze phospholipid to phospholipid acid (PA). *GhPLDα1* was highly expressed in the 20-DPA fibers, which may lead to a decrease in the phospholipid content and an increase in PA in 20-DPA fibers [41]. Consistently, most PA was enriched in the 20-DPA fibers. On the other hand, phospholipid acid can promote the production of hydrogen peroxide and induce the synthesis of SCW [41]. These results indicate that phospholipids play some roles in the regulation of fiber elongation and SCW synthesis. The intensity of saccharlipid (SL), sterol ester (ST), and wax ester (WE) was enriched at the stage of SCW deposition, which indicated that the enrichment of these lipid components may promote cellulose synthesis and SCW formation.

Phytosterols play important roles in the development of fiber cells [42,43,44,45]. Sterols are comprised of free sterols and conjugated sterols such as sitosterol ester (SiE), campesterol ester, stigmasterol (StE) ester, sterol glycosides (SGS), and acetyl sterol glycosides (ASGS). Conjugated sterols play a role in the dynamic balance of sterols and in the synthesis of WEs [46]. Three lipid classes of steryl esters, SiE, AGlcSiE, and StE, were detected in the study, among which, the AGlcSiE intensity was the highest in the rapid elongating fibers, and the intensity of SiE significantly increased in the stage of SCW synthesis. It was suggested that SiE might be associated with cellulose synthesis and SCW formation. Sterol glycosides and acetylsterol glycosides are enriched in elongating fibers. They may play roles in maintaining the balance between sterols and sphingolipids in fiber cell elongation [46,47]. Sterols and sphingolipids are two key components of lipid rafts, which are functional regions of membranes [48,49,50].

Sphingolipids are necessary for plant growth and development, in response to biotic stress or abiotic stress [51,52,53,54]. Sphingolipids also played a role in cotton fiber elongation. FB1 (Fumonisin B1), an inhibitor of sphingolipid synthesis, significantly inhibited fiber elongation and altered the activity of lipid raft activity in fiber cells [30,32]. In this study, CerG1, CerG2, CerG1 (d18:2/18:0), CerG1 (d18:2/18:1), CerG1 (d36:2), and CerG2 (d34:4) are enriched in the elongating fiber cells. Moreover, these molecules are closely associated with the AGlcSiE lipid molecule (Figure S3), which indicated that these molecules play a role in the regulation of fiber cell elongation. On the other hand, VLCFA is a component of sphingolipid molecules and plays an important role in fiber cell elongation. Application of ACE inhibited fiber elongation while VLCFA promoted cell elongation by promoting ethylene synthesis [22,23,55]. These results indicated that there is a close relationship between sphingolipids, sterol, and membrane lipid rafts during the process of fiber elongation. The future study of these relationships may be an important aspect to reveal the regulatory mechanism in the growth and development of fibers.

### 3.2. The Lipid Metabolism Disruption in the li-1 Mutant Fibers

*Ligon lintless-1* (*li-1*) is a dominant mutant of *Gossypium hirsutum*, which has the phenotype of damaged vegetative growth and short and thick fibers. Although the gene identification and gene expression profile of the fibers of the *li-1* mutant have been studied for many years, the regulatory mechanism of the fiber growth deficiency is still unclear [7,9,55]. Compared with the wild type, unexpectedly, most lipid classes and lipid molecule species have a higher rather than lower intensity in mutant fibers (20/25 lipid species, 60/68 lipid molecular species), and only 5 lipid species and 8 lipid molecule species are lower in mutant fibers. In the fibers of the *li-1* mutant, the lipid classes and lipid molecule species of TG and SL were enriched. Consistently, GL and SL were enriched in the stage of SCW synthesis in the wild-type fibers. The high intensity of GL and SL in the *li-1* fiber may promote SCW synthesis and inhibit fiber elongation. Consequently, the *li-1* fiber is shorter and thicker than the wild-type fiber. Sphingolipids and GPs were the major lipids in *li-1* cells. LPC and PLE were enriched in the 10-DPA fibers during fiber development in the wild type but decreased in the *li-1* fiber cells. These two GPs may play a key role in fiber elongation.

FB1 is an inhibitor of ceramide synthase that is the center of sphingolipid metabolism and biology [56,57]. The exogenous application of FB1 strongly inhibited fiber cell growth and its fiber phenotype is similar to that of the *li-1* fiber [32]. FB1 treatment resulted in a decrease in the total GIPC and all GIPC molecular species [32]. Consistent with the FB1 treatment, the total GIPC and all molecular species were reduced in the *li-1* mutant fibers (Figure 5F). Furthermore, the expression levels of two genes encoding ceramide synthases Gh_D12G0217 and Gh_A07G0513 were significantly reduced (Figure 8). These results revealed that GIPC synthesis was inhibited in the *li-1* fibers and GIPC might play an important role in fiber cell elongation. There are 10 homologous genes encoding ceramide synthase in the upland cotton genome. In our previous studies, overexpression of GhCS1 (Gh_D07G0583) promoted the synthesis of ceramide molecules containing dihydroxy LCB and VLCFA and inhibited the initiation and elongation of fiber cells [35]. Consistently, the most ceramide molecules containing dihydroxy LCB and VLCFA were significantly increased in the *li-1* mutant fibers (Figure 5D). Taken together, given sphingolipids are regarded as major regulators of lipid metabolism [58], sphingolipid balance might be an important factor for the normal growth of fiber cells.

During fiber development, SiE was enriched in the 20-DPA fibers while acetylglycosyl sterol ester and StE were enriched in the 10-DPA fibers. However, the lipid intensity of three sterol esters in the mutant fiber was higher than that in the wild-type fiber, and the intensity of the SiE molecule (SiE (18:3)) was 59.56 times that in the wild-type 10-DPA fibers. This change was further confirmed by the targeted lipidomics of phytosterols. Compared with TM-1, the contents of campesteryl ester, sitosteryl ester, and total steryl ester were strikingly increased in the *li-1* fibers by 21.8-, 48.7-, and 45.5-fold, respectively (Figure 6B). In plant cells, too high or too low levels of sterols will inhibit plant growth, and sterol esters play a role in regulating the level of free sterols [59]. The abnormal enrichment of sterol esters in the *li-1* fibers may be a reason for their elongation suppression.

Lipid droplets (LDs), also known as oil bodies or lipid bodies, are the “youngest” cellular organelle and are composed of a neutral lipid core surrounded by a phospholipid monolayer draped with hundreds of different proteins [60,61,62]. Since the hydrophobic core of LDs is mainly composed of TG and steryl esters [39,40,63], we further investigated the LDs in the leaf and fiber cells. The number and size of oil bodies in the *li-1* mutant obviously differ from those of TM-1. There were more and bigger oil bodies in both the leaf and fiber cells. LD biogenesis and degradation, as well as their interactions with other organelles, are tightly coupled to cellular metabolism and are critical to buffer the levels of toxic lipid species. Thus, LDs facilitate the coordination and communication between different organelles and act as vital hubs of cellular metabolism [39,40,64]. The change in the LDs further confirmed the disruption of the lipid metabolism in the fiber cells of *li-1*. This provided a novel clue to reveal the regulatory mechanism of fiber growth and development in the future.

## 4. Materials and Methods

### 4.1. Plant Materials and Growth Conditions

Gosspium hirsutum *ligon lintless-1* (*li-1*) mutant [7] and its wild-type Texas Marker-1 (TM-1) were kindly provided by the institute of Cotton Research, Chinese Academy of Agricultural Science, and were cultivated in a field with regular management. Flowers were tagged on the day of anthesis (0 DPA, days post-anthesis).

### 4.2. RNA Extraction and qRT-PCR Assay

The total RNA of 10-DPA and 20-DPA fiber cells from TM-1 and li-1 mutant plants was extracted by using a Plant Total RNA Extraction kit (Tiangen, Beijing, China). An amount of 1.0 μg total RNA was used to synthesize cDNA using a Reverse Transcription Kit with Genomic DNA Remover (Takara, Kusatu, Japan). Quantitative real-time reverse transcription PCR (RT-qPCR) was performed on a CFX96 Optical Reaction Module (Bio-Rad, Hercules, CA, USA) using Novostar-SYBR Supermix (Novoprotein, Shanghai, China) according to the manufacturer’s instructions. The primers for each gene were indicated in Supplementary Table S1. Cotton *GhHISTONE3* was used as an internal control. Their sequences are in the primer list in Supplementary Table S1. Each analysis was repeated with three biological replicates. Calculation method adopts 2^−ΔΔCt^.

### 4.3. Sample Preparation and Lipid Extraction and Lipidomics

The 10-DPA and 20-DPA fibers of the TM-1 (wild type) and the 10-DPA fibers of the *li-1* mutant were isolated from ovules and frozen in liquid N2. Lipids were extracted according to the MTBE (methyl tert-butyl ether) method. Briefly, samples were ground into fine powder in liquid nitrogen. The 100 mg samples were homogenized with 200 μL water and 240 μL precooled methanol. Then, 800 μL of MTBE was added and the mixture underwent ultrasound for 20 min at 4 °C followed by sitting still for 30 min at room temperature. The solution was centrifuged at 14,000× *g* for 15 min at 10 °C and the upper organic solvent layer was obtained and dried under nitrogen stream. The dried extraction was dissolved in 200 μL isopropanol by vortexing for 2 min and followed by centrifugation at 14,000× *g* for 15 min at 10 °C and the upper organic solvent layer was obtained and dried under nitrogen stream. The dried extraction was dissolved in 200 μL isopropanol by vortexing for 2 min and followed by centrifugation at 14,000× *g* for 15 min at 10 °C. The supernatant was subjected to analysis. Lipid analysis was performed using a UHPLC-MS/MS system consisting of a Shimadzu Nexera UHPLC system (manufactured by Shimadzu Corporation, Kyoto, Japan). Reverse-phase chromatography using a CSHC18 column (1.7 μm, 2.1 mm × 100 mm, Waters (Waters Corporation, Milford, MA, USA)) was chosen for LC separation. The lipid extracts were re-dissolved in 200 μL 90% isopropanol–acetonitrile, centrifuged at 14,000× *g* for 15 min, and finally, 3 μL of sample was injected. Solvent A was acetonitrile–water (6:4, *v*/*v*) with 0.1% formic acid and 0.1 Mm ammonium formate and solvent B was acetonitrile–isopropanol (1:9, *v*/*v*) with 0.1% formic acid and 0.1 mM ammonium formate. The initial mobile phase was 30% solvent B at a flow rate of 300 μL.min^−1^. It was held for 2 min, and then linearly increased to 100% solvent B in 23 min, followed by equilibrating at 5% solvent B for 10 min. Mass spectra were acquired by Q-Exactive Plus in positive and negative mode, respectively. ESI parameters were optimized and preset for all measurements as follows: source temperature, 300 °C; capillary temp, 350 °C; the ion spray voltage was set at 3000 V; S-Lens RF Level was set at 50%; and the scan range of the instruments was set at m/z 200–1800. “Lipid Search” is a search engine for the identification of lipid species based on MS/MS math. LipidSearch contains more than 30 lipid classes and more than 1,500,000 fragment ions in the database. Both mass tolerance for precursor and fragment were set to 5 ppm. OPLS-DA was applied to the data by using SIMCA-P Software 14.1 (Umetrics, Umeå, Sweden) to discover the lipid differences. The screening criteria were variable importance for the projection (VIP) > 1 and *p* value < 0.05.

### 4.4. The Nile Red Stain

Fresh fibers and leaves were placed in 2 mL EP tubes, fixed with paraformaldehyde, and placed at 4 °C for at least 4 h, overnight: washed twice with PBS (phosphate), dyed with 2 mL Nile red for about 1 h (tinfoil shielded from light), washed twice with PBS, and stored in PBS at 4 °C for later use.

### 4.5. Bioinformatic Analysis

Refer to the RNA-seq analysis method. Total RNA was extracted from the 10-DPA fiber cells of the *li-1* mutant and its wild-type TM-1. They were frozen in liquid nitrogen and sent to Shanghai Meiji Biological Company for Illumina Sequencing, meiji biological cloud web site (https://cloud.majorbio.com/ (accessed on 6 January 2024)) in the United States for the sequencing analysis results. The DEGseq difference Different analysis was used, at a significance level p-adjust difference multiple ≥2, multiple test correction method BH (FDR) with Correction with Benjamini/Hochberg, Differential Expression was obtained Genes), and then functional annotation analysis and functional enrichment analysis were performed on the obtained differentially expressed genes.

### 4.6. Statistical Analysis

All investigations were performed using three or six separate biological replicates. The results were presented as mean ± standard error (SE). A one-way analysis of variance was performed using SPSS 22 (IBM, New York, NY, USA) to determine significant differences. Statistical discrepancies were with *p*-values.

## 5. Conclusions

This study analyzed the lipid changes in the 10-DPA and 20-DPA fiber cells of wild-type (TM-1), as well as the lipid differences in the 10-DPA fibers between the TM-1 and *li-1* mutant. The predominant lipid classes enriched in fiber cells are GP (LPC, PA, PC, PE, PG, PI, and PS), SP (So, Cer, CerG1, CerG2), GL (DG and TG), SL (MGDG), ST (AGlcSiE), and Co. Compared to the rapid elongation stage of fibers, all SPs and most GPs, TG, and AGlcSiE were reduced, while CL LPA, MG, DGMG, MGMG, SiE, and WE increased in the secondary cell wall deposition stage of fibers. Dramatically, the contents of most lipid classes in the 10-DPA fibers were far higher in the *li-1* mutant than in TM-1, among which, the content of all STs strikingly increased, especially SiE and StE, which were increased by 64.37 and 3.99 times, respectively. Only LPC, LPE, CerG2, SM, and FA were decreased in the li-1 fiber cells. The detailed analysis of the sphingolipid and sterol profile showed that the contents of S1P, Cer, and GluCer significantly increased while those of Sph and GIPC drastically decreased in the *li-1* fibers. Similarly, the content of all sterols and conjugated sterols significantly increased in the *li-1* fibers, especially the content of SiE and total steryl esters, which increased by 48.7 and 45.5 times, respectively. The results from the transcriptomes showed that differentially expressed genes were enriched in the pathways of the lipid metabolism. Furthermore, the number and size of lipid droplets were altered in the *li-1* fiber cells and leaves. These results revealed that the lipid metabolism was disrupted drastically in the *li-1* short fiber cells and fiber cell elongation was blocked in the *li-1* mutant, which was not due to a lack of lipids, but rather lipid over-accumulation (obesity). This provides a new insight into the regulatory mechanism of fiber cell growth and development.

## Data Availability

Data are contained within the article.

## Supplementary Materials

The following supporting information can be downloaded at: https://www.mdpi.com/article/10.3390/ijms26031375/s1.

## Abbreviations

DPA, days post-anthesis; SCW, secondary cell wall; GP, glycerophospholipid; SP, sphingolipid; GL, glycerolipid; ST, sterol lipids; PL, prenol lipid; FA, fatty acid; SL, saccharolipid; CL, cardiolipin; Co, coenzyme; LPA, lysophosphatidic acid; LPE, lysophosphatidylethanolamine; LPI, lysophosphatidylinositol; PA, phosphatidic acid; PE, phosphatidylcholine; PG, phosphatidylglycerol; PI, phosphatidylinositol; PS, phosphatidylserine; Cer, ceramides; CerG1, glucocerebroside; SM, sphingomyelin; So, sphingosine; MG, monoglyceride; DG, diglyceride; TG, triglyceride; MGMG, Monogalactosylmonoacylglycerol; MGDG, monogalactosyldiacylglycerol; DGMG, Digalactosylmonoacylglycerol; DGDG, digalactosyldiacylglycerol; SQDG, Sulfoquinovosyldiacylglycerol; AGlcSiE, AcylGlcSitosterol ester; SiE, sitosterol ester; StE, stigmasterol ester; OAHFA, (O-acyl)-1-hydroxy fatty acid; WE, wax esters; ASG, acylated sterol glycoside; PLD, phospholipase D; SG, sterol glycoside; CerP, ceramides phosphate; phSM, phytosphingosine; FB1, Fumonisin B1; ACE, 2-chloro-*N*-[ethoxymethyl]-*N*-[2-ethyl-6-methyl-phenyl]-acetamide; VLCFA, very-long-chain fatty acids; PC, phosphatidylserine; PS, phosphatidylserine; PG, phosphatidylglcholine; PhytoCer, phytoceramides; GluCer, glucosylceramides; GIPC: glycosyl inositol phospho ceramides; S1P: sphingosine-1-phosphate; Phyto-GluCer, phyto-glucosylceramides; PhytoSph, phytosphingosines; PhytoCer-OHFA, phytoceramides with hydroxylated fatty acyls; LD: lipid droplets; LPC, lysophosphatidylcholine.
