# Supplementary material for: The Disruptions of Sphingolipid and Sterol Metabolism in the Short Fiber of Ligon-Lintless-1 Mutant Revealed Obesity Impeded Cotton Fiber Elongation and Secondary Cell Wall Deposition
Source: Int J Mol Sci. 2025 Feb 6;26(3):1375. doi: 10.3390/ijms26031375 (PMC11818067; doi:10.3390/ijms26031375)
Supplement: Supplementary file 1 [file ijms-26-01375-s001.zip › Supplementary Captions.pdf]

Figure S1. The volcano plot and hot map for the different lipid molecule species between 20-DPA fibers and 10-DPA fibers from wild type (TM-1)

A: The volcano plot. The horizontal ordinate represents the differential accumulation fold value after log2 transformation, and the Y-axis represents the p-value after log10 transformation. The dots represent lipid molecule species and the rose red dots are lipid molecule species that meet the screening criteria of differential accumulation fold (FC > 2 or FC < 0.5, p-value < 0.05). B: Hierarchical clustering analysis of the different lipid molecule species between 20-DPA fibers and 10-DPA fibers from wild type (TM-1). The horizontal ordinate represents the samples and the Y-axis represents the differential lipid molecule species. The color is related to the lipid intensity of lipid molecule species. Lipid molecule species with similar accumulation patterns were clustered in the same cluster on the left.

Figure S2. The volcano plot and hot map for the different lipid molecule species between 10-DPA fibers from wild type (TM-1) and 10-DPA fibers from *Li-1* mutant

A: The volcano plot. The horizontal ordinate represents the differential accumulation fold value after log2 transformation, and the Y-axis represents the p-value after log10 transformation. The dots represent lipid molecule species and the rose red dots are lipid molecule species that meet the screening criteria of differential accumulation fold (FC > 2 or FC < 0.5, p-value < 0.05). B: Hierarchical clustering analysis of the different lipid molecule species between 10-DPA fibers of TM-1 and 10-DPA fibers from *Li-1* mutant. The horizontal ordinate represents the samples and the Y-axis represents the differential lipid molecule species. The color is related to the lipid intensity of lipid molecule species. Lipid molecule species with similar accumulation patterns were clustered in the same cluster on the left.

Figure S3. Correlation Analysis of different lipid molecule species in three samples

FigureS4. The heat map of different lipid molecule species in TM-1 and *Li-1* mutant

FigureS5. Heat map of gene cluster analysis

Each column in the figure represents a sample, each row represents a gene, and the color in the figure represents the expression value of the gene after standardized treatment in each sample. Red represents the higher expression level of the gene in the sample, and blue represents the lower expression level. For the specific change trend of the expression level, please see the digital annotation under the color bar on the upper left. The left side is the tree diagram of gene clustering and the module diagram of sub-clustering, and the right side is the name of gene. The closer the two gene branches are, the closer their expression levels are. The top is a tree diagram of sample clustering, and the bottom is the names of 10-DPA fibers of TM-1 and 10-DPA fibers from *Li-1* mutant samples, respectively. The closer the branches of two samples are, the closer the expression patterns of all genes in the two samples are, that is, the closer the change trend of gene expression quantity is.

Data S1. The lipid intensity of lipid classes in three samples

Data S2. The data of lipidomics of three samples

Table S1. The sequence of primer used to detect expression level in RT-qPCR
