# Supplementary material for: The Disruptions of Sphingolipid and Sterol Metabolism in the Short Fiber of Ligon-Lintless-1 Mutant Revealed Obesity Impeded Cotton Fiber Elongation and Secondary Cell Wall Deposition
Source: Int J Mol Sci. 2025 Feb 6;26(3):1375. doi: 10.3390/ijms26031375 (PMC11818067; doi:10.3390/ijms26031375)
Supplement: Supplementary file 1 [file ijms-26-01375-s001.zip › Table S1.pdf]

Table S1. The sequence of primer used to detect expression level in RT-qPCR

Primer	Sequence(5'-3)
qPCR -0217-F	ATTTTCTCGTGGCACTTTAC
qPCR -0217-R	AACCATGGCTCGTAGTAGGT
qPCR-0513-F	TGGCTTGGAATGGCTTGCTA
qPCR -0513-R	AACGACACGCGTAGAATGGT
qPCR -0211-F	ATCCTTCCCGGTGACAAACC
qPCR -0211-R	TGGACTGTGCACCAATGACA
qPCR -0144-F	ACGCCGTTGGTGGATGTATT
qPCR -0144-R	GGGTGACCTAAGATGCCGAC
qPCR -0884-F	CGCTGGAATCCTTGACGAGA
qPCR -0884-R	CTGTCCTGTGGCTCTTTGGT
qPCR-1074-F	CCTCTTTGCTGCTGATGGGA
qPCR-1074-R	CCAATTTGACCGCCTGTTGG
qPCR -1600-F	ATGAAGAGACCCGCCGTTTT
qPCR -1600-R	CGTGAAGCAAGCGTTGTGAA
qPCR -1605-F	ACTGCCGGTCAGGTTATTCG
qPCR -1605-R	GGCCAACATGGACGACAGTA
qPCR-2376-F	CTGCGAAAGCCGTAACGATG
qPCR -2376-R	AGCCCTAGACTTCGTCCCAT
qPCR -3810-F	CGGTTGCAGGAACTTGTGTG
qPCR -3810-R	GGTCTACTGCTATGGCGACC
qPCR-His-F	CCGTCCTGGAACTGTTGCTCT
qPCR-His-R	ACCCACAAGGTATGCCTCTGC
