# Supplementary figures and images for: The Disruptions of Sphingolipid and Sterol Metabolism in the Short Fiber of Ligon-Lintless-1 Mutant Revealed Obesity Impeded Cotton Fiber Elongation and Secondary Cell Wall Deposition
Source: Int J Mol Sci. 2025 Feb 6;26(3):1375. doi: 10.3390/ijms26031375 (PMC11818067; doi:10.3390/ijms26031375)

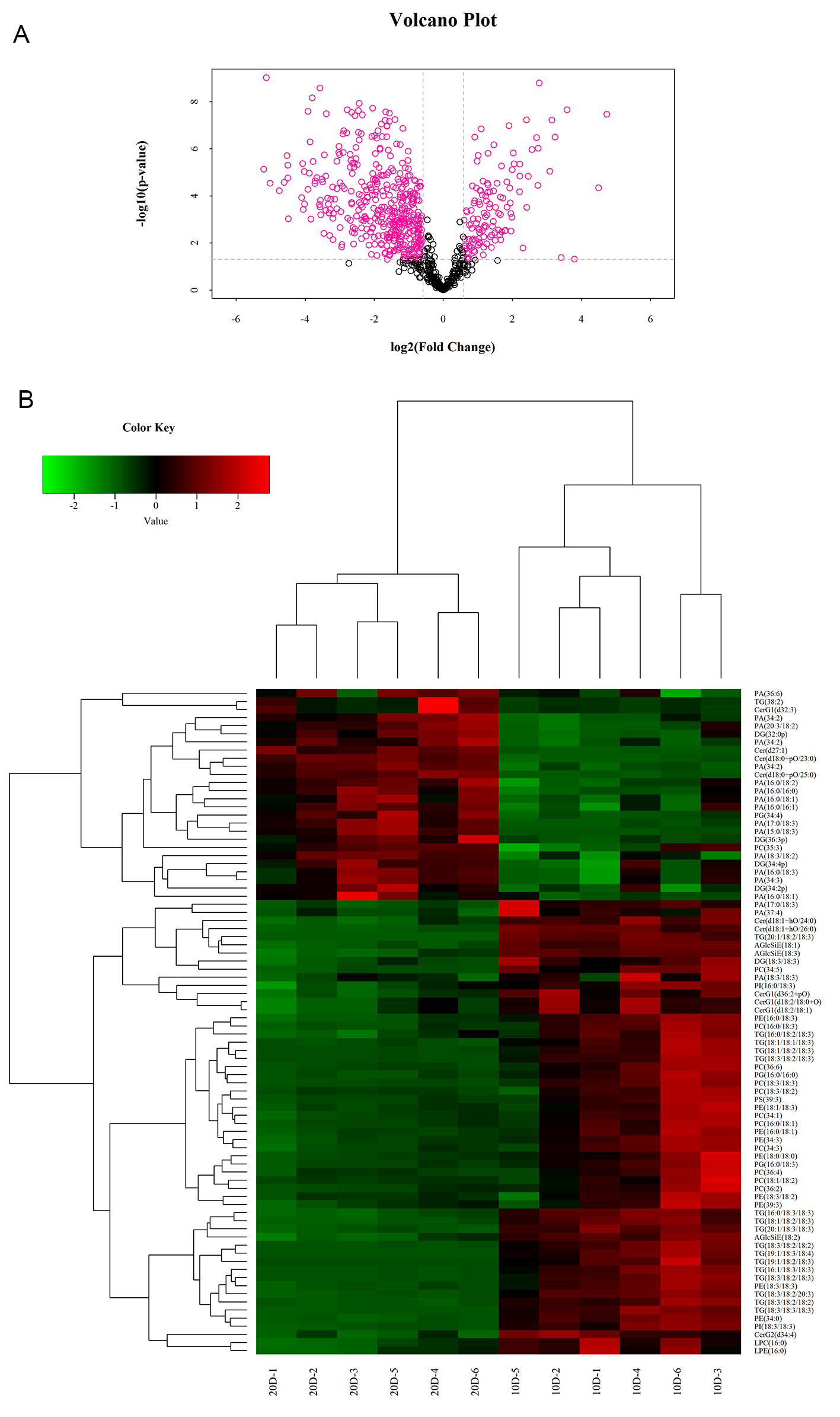

Supplement: Supplementary file 1 [file ijms-26-01375-s001.zip › Figure S1(20D Vs 10D).jpg]

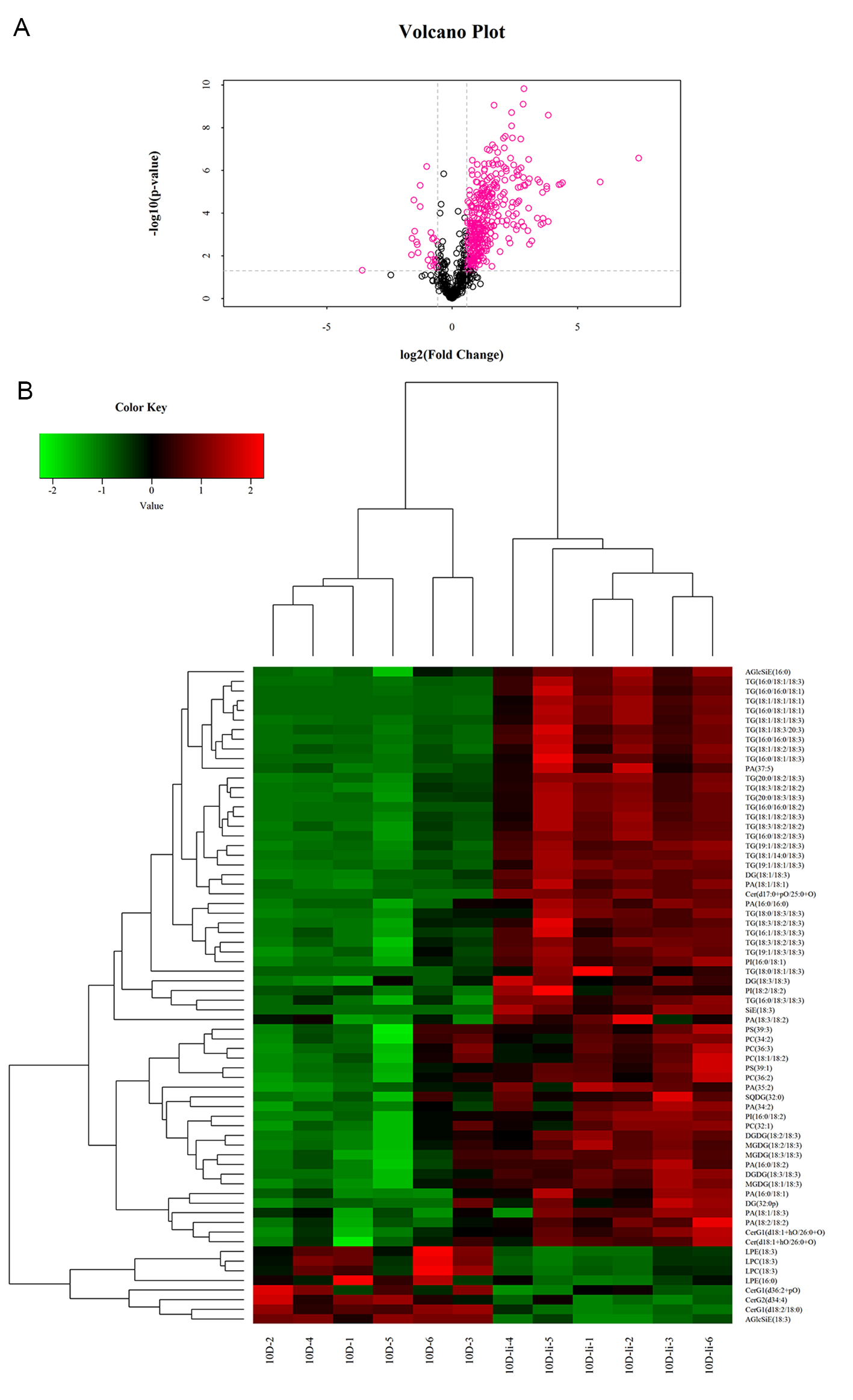

Supplement: Supplementary file 1 [file ijms-26-01375-s001.zip › Figure S2(10D-li Vs 10D).jpg]

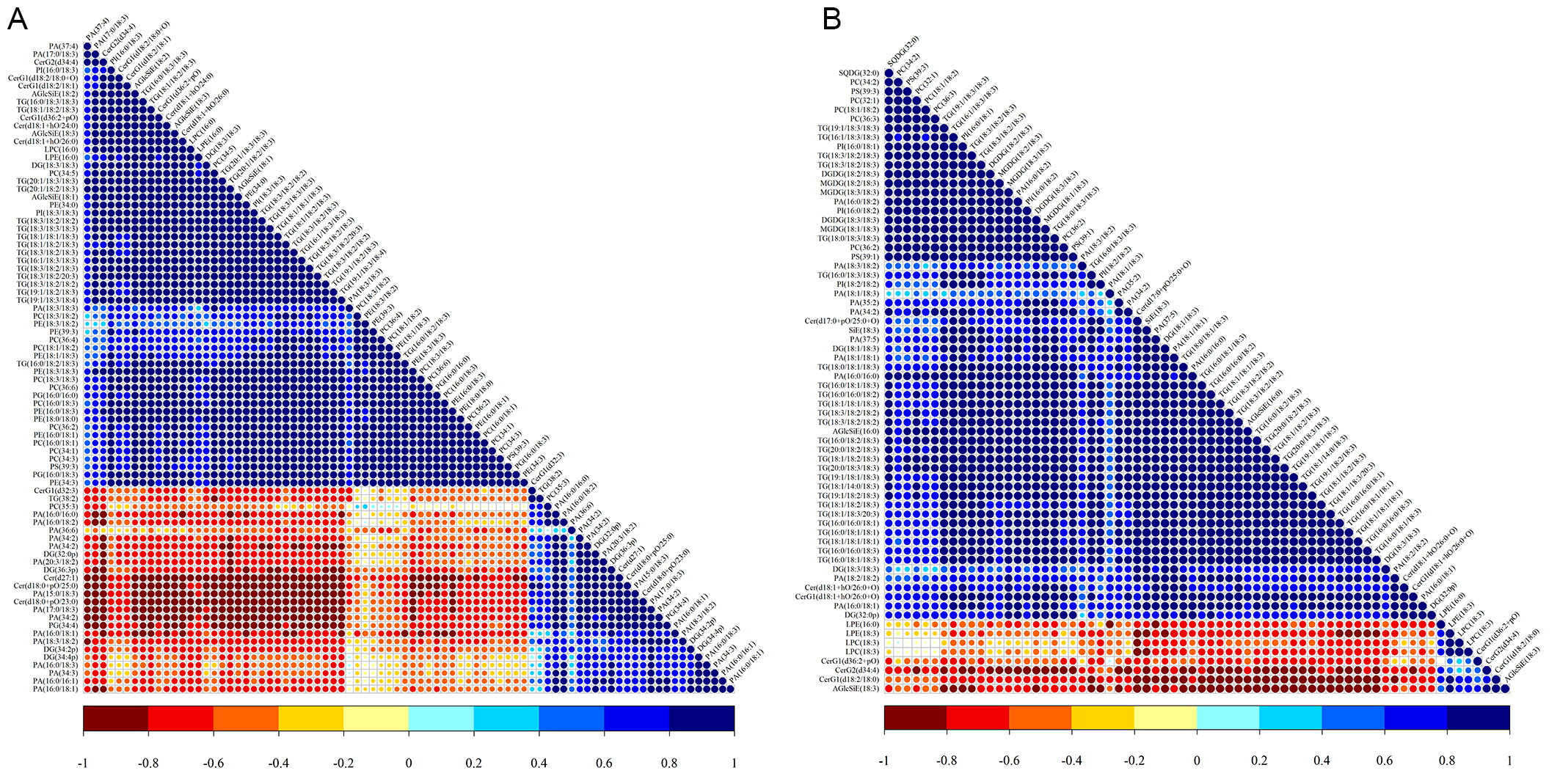

Supplement: Supplementary file 1 [file ijms-26-01375-s001.zip › Figure S3.jpg]

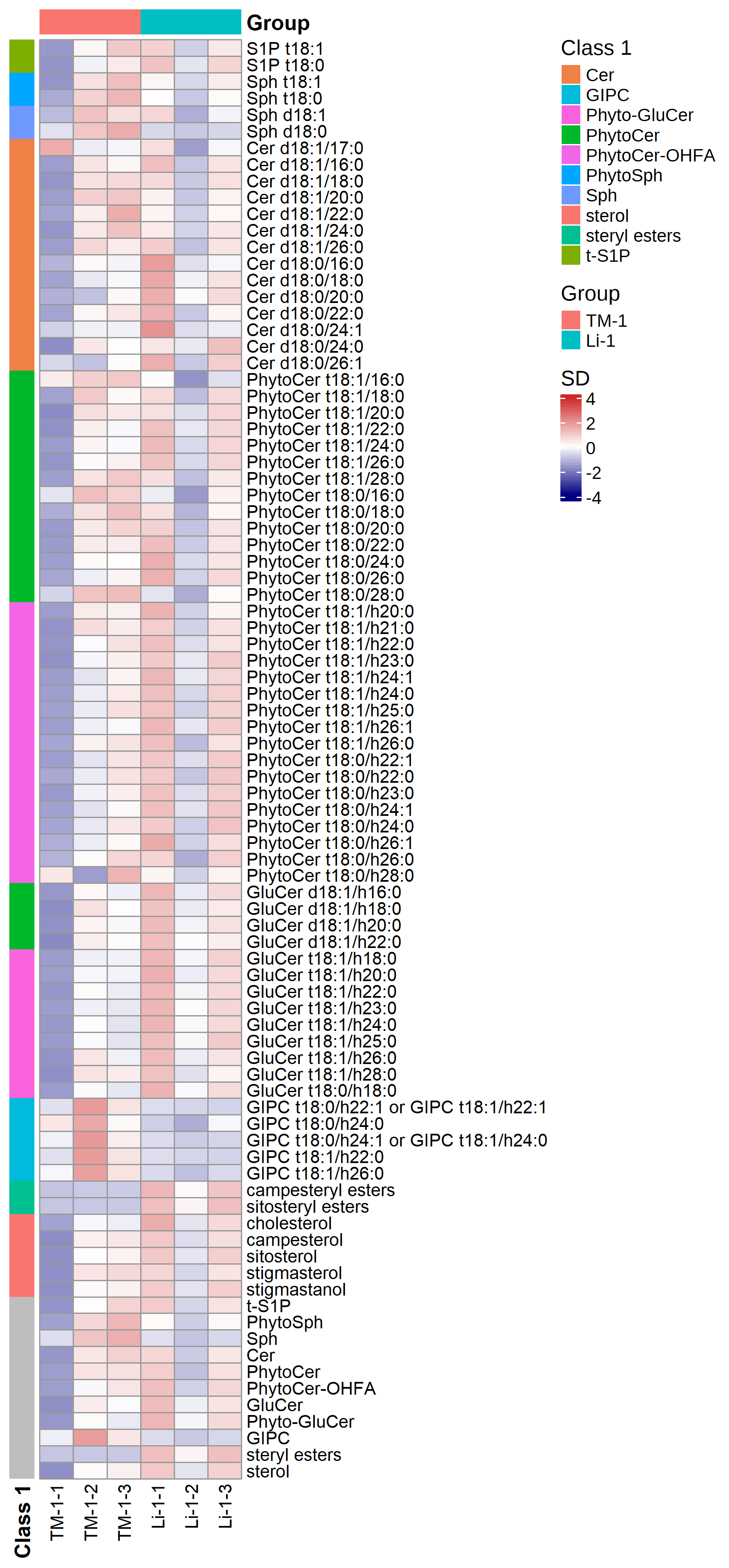

Supplement: Supplementary file 1 [file ijms-26-01375-s001.zip › Figure S4.tiff]

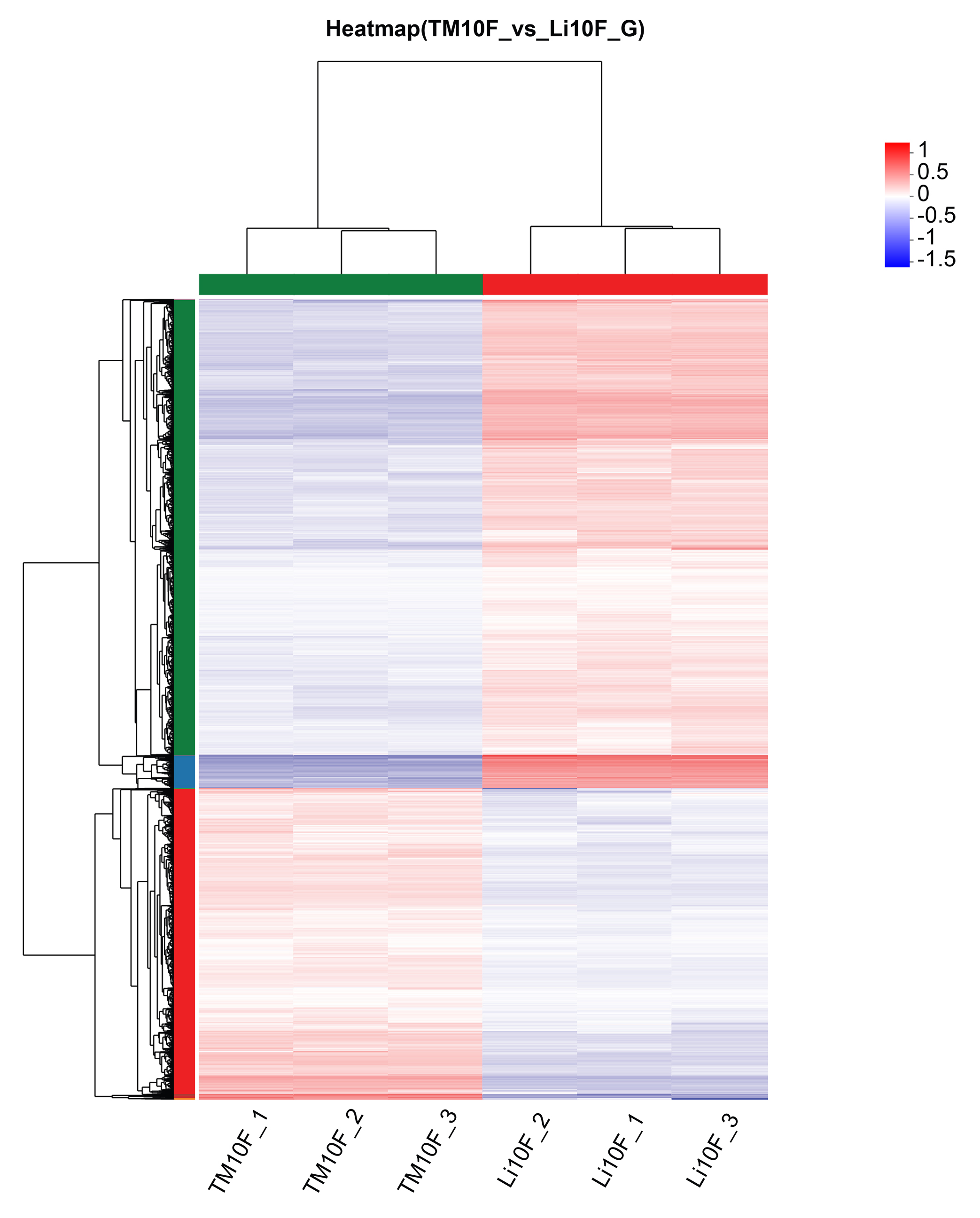

Supplement: Supplementary file 1 [file ijms-26-01375-s001.zip › Figure S5.jpg]
